# Impact of blastocyst grading and blastocyst biopsy dates on the clinical outcomes of patients undergoing preimplantation genetic testing

**DOI:** 10.3389/fendo.2024.1427922

**Published:** 2024-09-20

**Authors:** Chang Tan, Xiliang Wang, Pengshu Zou, Wei Wei, Li Yan, Kaiyue Wang, Yuexin Yu

**Affiliations:** Department of Reproductive Medicine, General Hospital of Northern Theater Command, Shenyang, China

**Keywords:** preimplantation genetic testing, clinical pregnancy outcome, assisted reproduction, blastocyst biopsy, blastocyst grading

## Abstract

**Background:**

Preimplantation genetic testing (PGT) allows for the evaluation of embryo genetic information prior to implantation, enabling the selection of normal embryos for transfer and ultimately leading to better pregnancy outcomes. In this study, we explored factors that influence clinical outcomes of patients undergoing PGT. The effects of blastocyst grading and biopsy dates on clinical outcomes were also analyzed.

**Methods:**

The clinical data and pregnancy outcomes of 428 PGT cycles performed in the Reproductive Medicine Department of the Northern Theater General Hospital between January 2017 and December 2022 were retrospectively analyzed. Multifactorial logistic regression analysis and nomograms were used to determine factors influencing pregnancy outcomes. The impact of D5 blastocysts (290 cycles) and D6 blastocysts (138 cycles) with different quality levels on clinical outcomes was also compared.

**Results:**

Multifactorial logistic regression analysis showed that age, BMI, endometrial thickness, and embryo quality of women affected PGT clinical outcomes. Women aged <40 years or with a body mass index (BMI) >18.5 and endometrial thickness>1.0 cm had a significantly higher pregnancy success rate. Compared to that of D6 blastocyst biopsy, D5 blastocyst biopsy was associated with a higher pregnancy success rate but a similar live birth rate. No significant differences were observed in the pregnancy and live birth rates of D5 and D6 high-quality blastocysts.

**Conclusion:**

To achieve better pregnancy outcomes after PGT, considering blastocyst grading and biopsy dates when transferring embryos is essential for improving pregnancy outcomes. Furthermore, patients should adjust their BMI, endometrial receptivity, and endometrial thickness and pattern.

## Introduction

Embryonic abnormalities (genetic or chromosomal) cause implantation failure and miscarriages (1, 2). The incidence of chromosomal abnormalities is high in early human embryos, regardless of whether they are produced naturally or through assisted reproductive technology (3). During female fertility, including the peak period, the incidence of chromosomal abnormalities cannot be ignored, affecting approximately 20% of women (4). Therefore, approximately half of human preimplantation embryos have chromosomal abnormalities (5–7), and this high incidence is the main cause of implantation failure, miscarriage, or the birth of offspring with potential abnormalities (8, 9). To improve clinical outcomes in patients struggling with infertility, identifying abnormal embryos before transfer is crucial. The development of preimplantation genetic testing (PGT) technology has made it possible to evaluate embryos and analyze their genetic information implantation into the uterus, allowing normal embryos to be selected for transfer and, ultimately, achieving a better pregnancy outcome. This technology has little impact on embryo viability but results in a higher implantation rate (10–13).

Embryonic biopsy is an important step in the process of PGT. The continuous development of PGT technology has made it possible to extract 5-10 trophoectoderm cells from blastocysts for biopsy and genetic testing. Biopsies are usually performed on blastocysts formed on D5 or D6; however, the effects of different developmental days and blastocysts quality on clinical outcomes remain unclear.

In this study, we aimed to analyze factors affecting the clinical outcomes of patients undergoing PGT, including age, body mass index (BMI), endometrial thickness, endometrial pattern, blastocyst development duration, and embryo quality. Our analysis was conducted from a clinical perspective. Furthermore, we compared the clinical outcomes of blastocysts with different biopsy dates and quality levels, encompassing high-quality blastocysts and poor-quality blastocysts. Here, advanced maternal age was defined as 37 years or older (14). We aimed to provide theoretical support for enhancing clinical pregnancy outcomes in patients undergoing PGT.

## Materials and methods

### Study design and patients

Retrospective analysis was performed using data from patients affected by infertility who underwent PGT for fertility treatment at our center from January 2017 to December 2022, encompassing a total of 428 PGT cycles, only the first transfer cycle was included in the study for each enrolled female patient. All patients underwent genetic counseling and signed informed consent forms before entering the PGT cycle. All experimental protocols were approved by the medical ethics committee of the General Hospital of Northern Theater Command, and all methods were carried out in accordance with the Declaration of Helsinki.

Inclusion criteria (any one of the following criteria was sufficient): Receiving PGT transfer for conception includes: (1) patients undergoing PGT-A due to advanced maternal age (≥37 years), unexplained repeated implantation failure: Failure of implantation in three or more attempts, or high-scoring cleavage-stage embryos or blastocysts that have all failed to implant, and history of recurrent pregnancy loss. (2) patients undergoing PGT-SR cycles because one person in the couple had chromosomal abnormalities (including reciprocal translocation, Robertsonian translocation, inversion, 47,XXX, and 47,XXY); and (3) one of the patients undergoing PGT-M for a single-gene disease. All 428 cycles included in the study were the patients’ first transfer cycles, which included 241 PGT-A cycles, 14 PGT-M cycles, and 173 PGT-SR cycles.

The exclusion criteria included the following conditions: untreated hydrosalpinx or tubal dilation; uterine cavity abnormalities such as untreated endometrial polyps, submucosal uterine myomas, and moderate to severe intrauterine adhesions, along with uterine malformations; untreated endometriotic cysts within the ovaries; and transfer of mosaic embryos.

### Data collection

The information on demographics and characteristics of enrolled patients was collected from the electronic medical record system; the information included the patient’s age, body mass index (BMI), infertility type, days of embryo transfer, embryo quality, endometrial thickness, and endometrial pattern on the day of transfer (A: a three-line pattern, with a visible high echogenicity central line; B: a transitional type, characterized by isolated echogenicity in the middle and indistinct echogenicity in the midline of the uterine cavity; the midline echogenicity of the uterine cavity is not obvious; A^-^: between A and B).

### Preimplantation genetic testing

Embryo biopsy was performed at the blastocyst stage when all embryos had reached sufficient expansion by the fifth or sixth day after fertilization. A small number of cells (5–10) were dissected from the trophectoderm cell mass that herniated after the laser-assisted hatching of the zona pellucida. The cells were then transferred to a PCR tube with 2 μl of phosphate buffered saline (PBS). Whole-genome amplification was performed. Normal embryos suitable for transfer were screened using next-generation sequencing, frozen, and transferred later. One euploid embryo was transferred per cycle.

### Blastocyst quality assessment

At least two experienced embryologists independently evaluated the blastocysts using the Gardner and Schoolcraft grading system (15). Three morphological parameters primarily determined the blastocyst score: blastocyst expansion, inner cell mass (ICM), and trophectoderm (TE). Quality levels were defined as follows: Blastocysts with an expansion stage of ≥4 and both ICM and TE grade scores of 4AA, 4AB, 4BA, or 4BB were classified as high-quality. Conversely, if either the ICM or TE score was a C, the blastocysts were classified as poor-quality, specifically with scores of 4BC, 4CB, 4AC, or 4CA (16). At our center, embryos were biopsied once blastocyst expansion reached stage 4; hence, embryos with a biopsy date on day 5 were categorized as D5 blastocysts, while those biopsied on day 6 were categorized as D6 blastocysts.

### Blastocyst transfer

Endometrial thickness was monitored by ultrasound on days 3–5 of the menstrual cycle, and 2 mg estradiol valerate was administered twice daily on the same day. This dose was adjusted based on the endometrial thickness every 4 days. When the thickness of the endometrium exceeded 8 mm, progesterone (40 mg/day) was injected intramuscularly, and blastocysts were transferred after 5 days of endometrial transformation. Luteal support was continued after transfer completion. The embryos were cultured for 4–5 h after resuscitation and then transferred.

### Pregnancy

On the 14th day after embryo transfer, serum human chorionic gonadotropin tests were measured. If positive, an ultrasound examination was performed 10–14 days later to confirm intrauterine pregnancy. Clinical pregnancy is defined as the identification of a gestational sac with fetal heart activity on an ultrasound examination. Live birth was defined as the delivery of one or more live babies beyond 28 weeks of gestation.

### Statistical methods

Data were analyzed using SPSS 25.0. These characteristics were assigned to continuous and categorical data. Continuous variables were expressed as x̅ ± s, and between-group comparisons were performed using the independent samples t-test. Categorical variables are expressed as n (%), and between-group comparisons were made using nonparametric chi-square tests. Univariate and multivariate logistic regression analyses were performed to compare relevant factors affecting clinical pregnancy rates. Odds ratios (OR) with 95% confidence intervals (CI) were calculated as effect estimates, clinical outcome-related variables were included in the Stepwise Akaike Information Criterion (STEP AIC) analysis to select factors for the establishment of a probability predictive nomogram, and the C-index was used to determine the clinical usefulness of the model. All reported P values were two-sided, with a significance level of 0.05.

## Results

### Baseline characteristics

In total, 428 PGT cycles were included in the final analysis. Of these, 263 cycles resulted in clinical pregnancies, while 163 did not result in pregnancy. Differences in the age (P=0.812), BMI (P=0.940), years of infertility (P=0.523), endometrial pattern (P=0.703), and type of infertility (P=0.726) between the two groups were not statistically significant. However, we noted significant differences between the two groups for endometrial thickness (*P=0.021*), embryo quality (*P<0.001*), and days of blastocyst development (*P=0.004*) (**Table 1**). To further reveal the association between the related factors and clinical pregnancy, we performed univariate and multivariate logistic regression analyses (**Table 2**). The result showed that the pregnancy outcomes of patients aged ≥40 years (OR 0.41; 95% CI 0.21–0.81) were significantly worse than those of patients aged <40 years, but the pregnancy outcomes of patients with BMI >23.9 (OR 2.53; 95% CI 1.09–5.88) and endometrial thickness >1.0 (OR 1.92; 95% CI 1.13–3.26) were significantly better than those of the other patients (*P<0.05*). In the multivariate logistic analyses, based on the OR (95% CI) and P-value results, embryo quality and days of blastocyst development were significantly correlated with pregnancy outcome; univariate logistic regression analysis yielded similar results (**Table 2**).

**Table 1:** Characteristics of patients with clinically defined pregnancy and no pregnancy after preimplantation genetic testing.

Characteristics	Total	Pregnancy Group (N=263)	No Pregnancy Group (N=165)	P-value
Age (years)	33.69 ± 4.30	33.38 ± 4.22	34.18 ± 4.39	0.812
BMI (kg/m^2^)	23.44 ± 3.72	23.58 ± 3.72	23.22 ± 3.71	0.940
Years of infertility	2.83 ± 2.44	2.82 ± 2.39	2.86 ± 2.52	0.523
Infertility type Primary Secondary				
	101 (23.60%)	64 (63.37%)	37 (36.63%)	0.726
	327 (76.40%)	199 (60.86%)	128 (39.14%)	
Endometrial thickness (cm)	0.93 ± 0.21	0.95 ± 0.22	0.90 ± 0.19	***0.021***
Endometrial pattern A A^-^ B				
	20 (4.67%)	14 (70%)	6 (30%)	0.703
	114 (26.64%)	71 (62.28%)	43 (37.72%)	
	294 (68.69%)	178 (60.54%)	116 (39.46%)	
Embryo quality High-quality Poor-quality				***<0.001***
	380 (88.79%)	246 (64.74%)	134 (35.26%)	
	48 (11.21%)	17 (35.42%)	31 (64.58%)	
Days of blastocyst development D5 D6				***0.004***
	290 (67.76%)	193 (66.55%)	97 (33.45%)	
	138 (32.24%)	70 (50.72%)	68 (49.28%)	

BMI, body mass index; Values were given as mean ± SD or n (percentage%). Differences in baseline characteristics were compared using χ^2^ tests for categorical variables and independent samples t-tests for continuous variables.

**Table 2:** Results of the univariate and multivariate logistic regression analysis for factors associated with pregnancy outcomes.

Characteristics	Univariable		Multivariable	
	OR (95% CI)	P value	OR (95% CI)	P value
Age (years) <37 37~39 ≥40	1.0 1.14 (0.68, 1.90) 0.41 (0.21, 0.81)	- 0.618 ***0.010***	1.0 1.11 (0.64, 1.90) 0.46 (0.23, 0.93)	- 0.717 ***0.031***
BMI (kg/m^2^) ≤18.5 18.5~23.9 >23.9	1.0 2.15 (0.95, 4.87) 2.53 (1.09, 5.88)	- 0.068 ***0.031***	1.0 2.45 (1.03, 5.80) 2.57 (1.05, 6.27)	- ***0.043*** ***0.039***
Infertility type Primary Secondary	1.0 0.90 (0.57, 1.43)	- 0.651	1.0 1.10 (0.65, 1.87)	- 0.728
Endometrial thickness(cm) ≥0.8 0.81~1.0 >1.0	1.0 1.22 (0.78, 1.90) 1.92 (1.13, 3.26)	- 0.385 ***0.016***	1.0 1.29 (0.81, 2.06) 1.96 (1.10, 3.52)	- 0.289 ***0.023***
Endometrial pattern A A^-^ B	1.0 0.71 (0.25, 1.98) 0.65 (0.24, 1.75)	- 0.510 0.398	1.0 0.95 (0.33,2.75) 0.80 (0.29,2.21)	- 0.926 0.666
Embryo quality High-quality Poor-quality	1.0 0.30 (0.16, 0.56)	- ***<0.001***	1.0 2.61 (1.32, 5.16)	- ***0.006***
Days of blastocyst development D5 D6	1.0 0.51 (0.34, 0.72)	- ***<0.001***	1.0 0.62 (0.39, 0.98)	- ***0.039***

BMI, body mass index; CI, confidence interval; OR, odds ratio.

### Establishment of a nomogram for predicting clinical outcomes of PGT cycles

**Figure 1:**
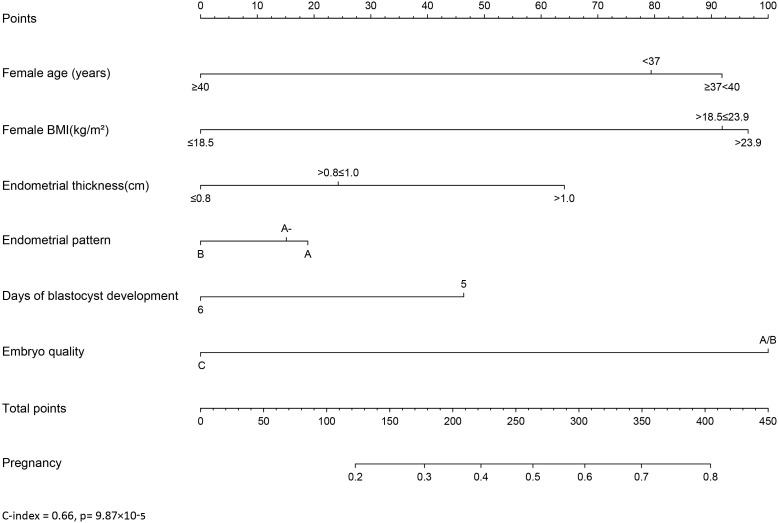
Nomogram to predict the probability of clinical pregnancy for PGT patients.

A nomogram was developed to provide a quantitative and convenient tool for predicting clinical pregnancy outcomes based on patient age, BMI, endometrial thickness, endometrial pattern, days of blastocyst development, and embryo quality (**Figure 1**), C-index = 0.66 (*p= 9.87×10^-5^)*. To estimate an individual’s incidence of clinical pregnancy, each variable was located, a vertical line was drawn, the corresponding score was determined, and the points from each variable value were summed. The higher the score, the greater the probability of clinical pregnancy, providing a personalized probability of clinical pregnancy for patients with PGT.

### Blastocyst biopsy on D5 and D6

**Figure 2:**
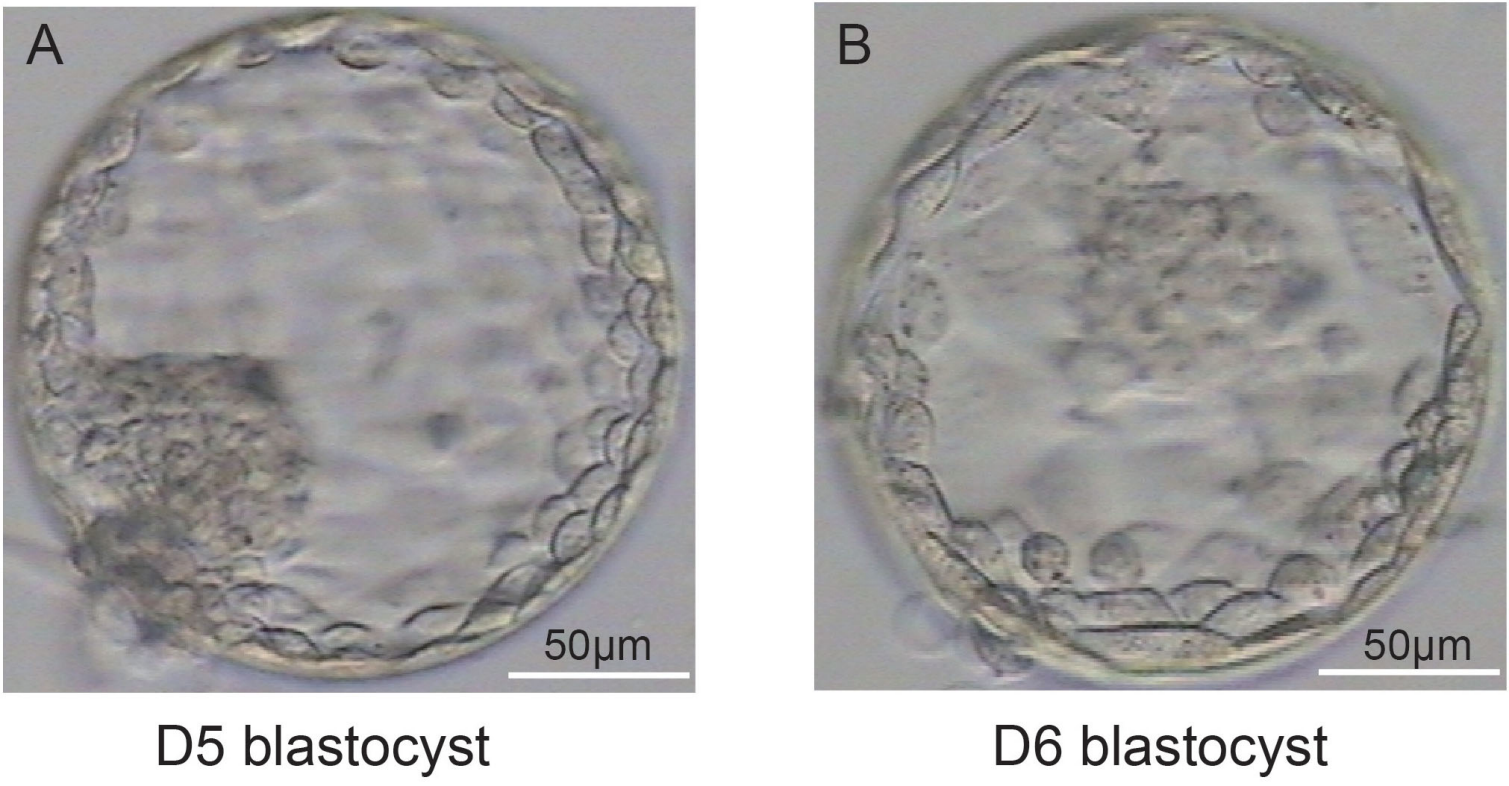
Representative images of blastocysts at D5 **(A)** and D6 **(B)**. **(A, B)** illustrate that the blastocysts have both met the criteria for a stage 4 blastocyst: the blastocoel completely occupies the total volume of the embryo, the total volume of the embryo increases, the zona pellucida becomes thinner.

Representative images of blastocyst biopsies obtained on D5 (**Figure 2A**) and D6 (**Figure 2B**) are shown in **Figure 2**. In our laboratory if the blastocysts of PGT patients had reached sufficient expansion and stage 4, combined with morphological analysis, the embryology lab staff would perform a biopsy. If a D5 blastocyst scored 4 or above, a biopsy was performed; for D5 blastocysts that did not achieve grade 4, we generally continued cultivation. A biopsy was performed on the D6 blastocyst that scored 4. D7 blastocysts that reached grade 4 were rare and were therefore excluded from this study.

### Comparison of the pregnancy and live birth rates of patients who received blastocyst transfer on D5 and D6

**Figure 3:**
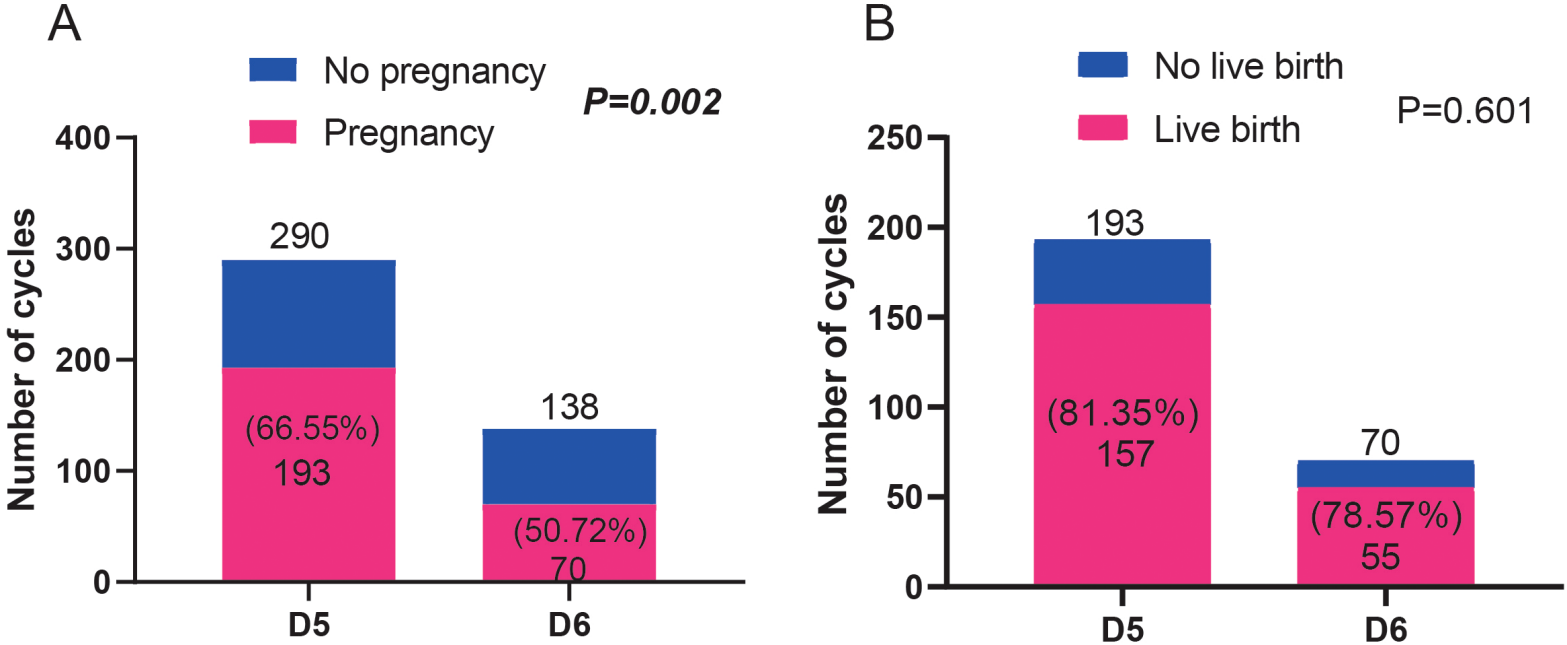
Comparison of the pregnancy ratio **(A)** and live birth ratio **(B)** between patients who received blastocyst transfer on D5 and D6. Fisher’s exact test was used for statistical analyses.

The clinical outcomes of D5 and D6 blastocyst transfer were analyzed. As shown in **Figure 3A**, the pregnancy rate in the D5 blastocyst transfer group was significantly higher than that in the D6 group (*P*=0.002). However, no differences in live birth rates were observed between D5 and D6 groups (*P*=0.601) (**Figure 3B**).

### Pregnancy and live birth rates of patients who received blastocyst transfer of different qualities on D5 and D6

**Figure 4:**
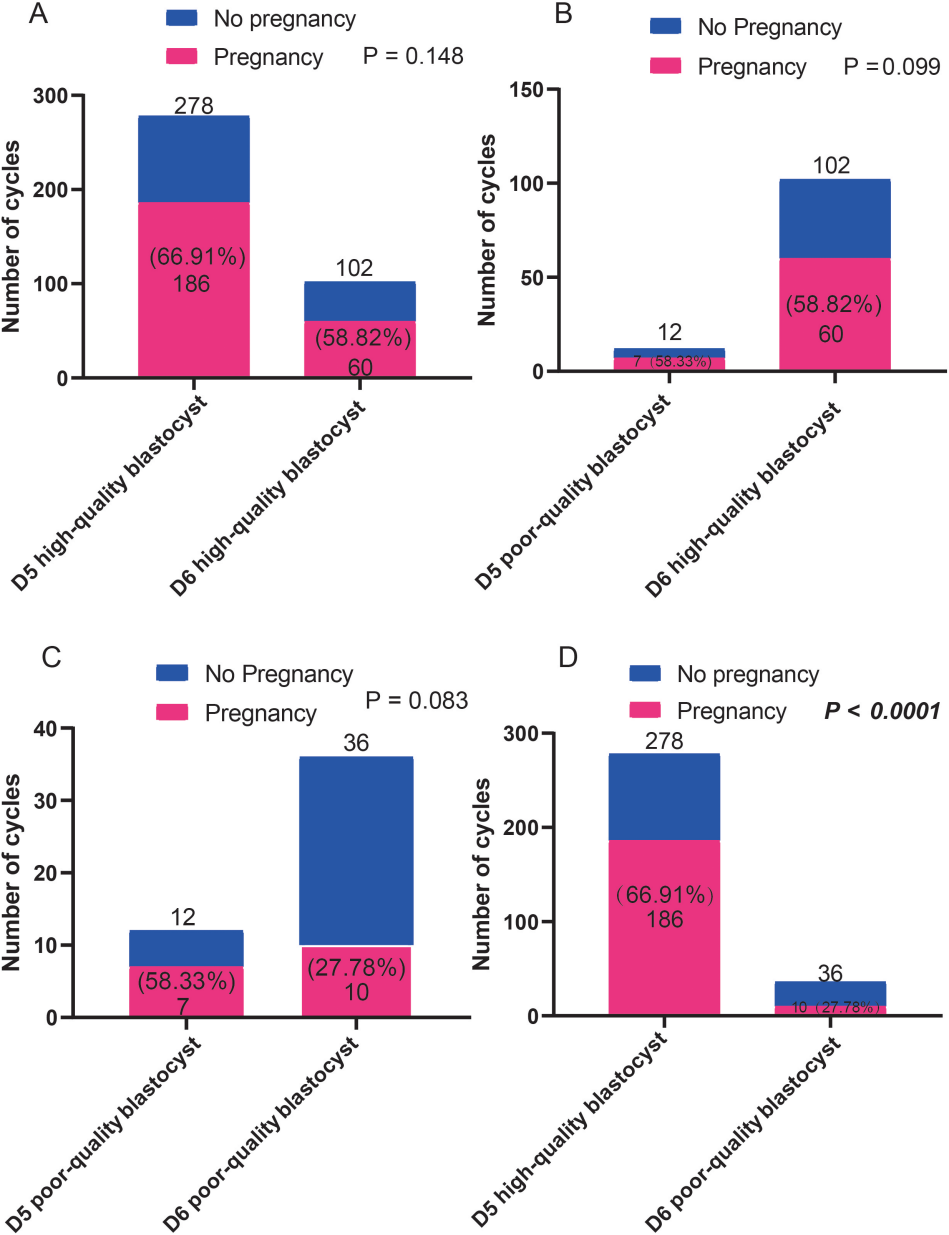
Comparison of the pregnancy ratios **(A-D)** between patients who received blastocyst transfer of different qualities on D5 and D6. Fisher’s exact test was used for statistical analysis.

**Figure 5:**
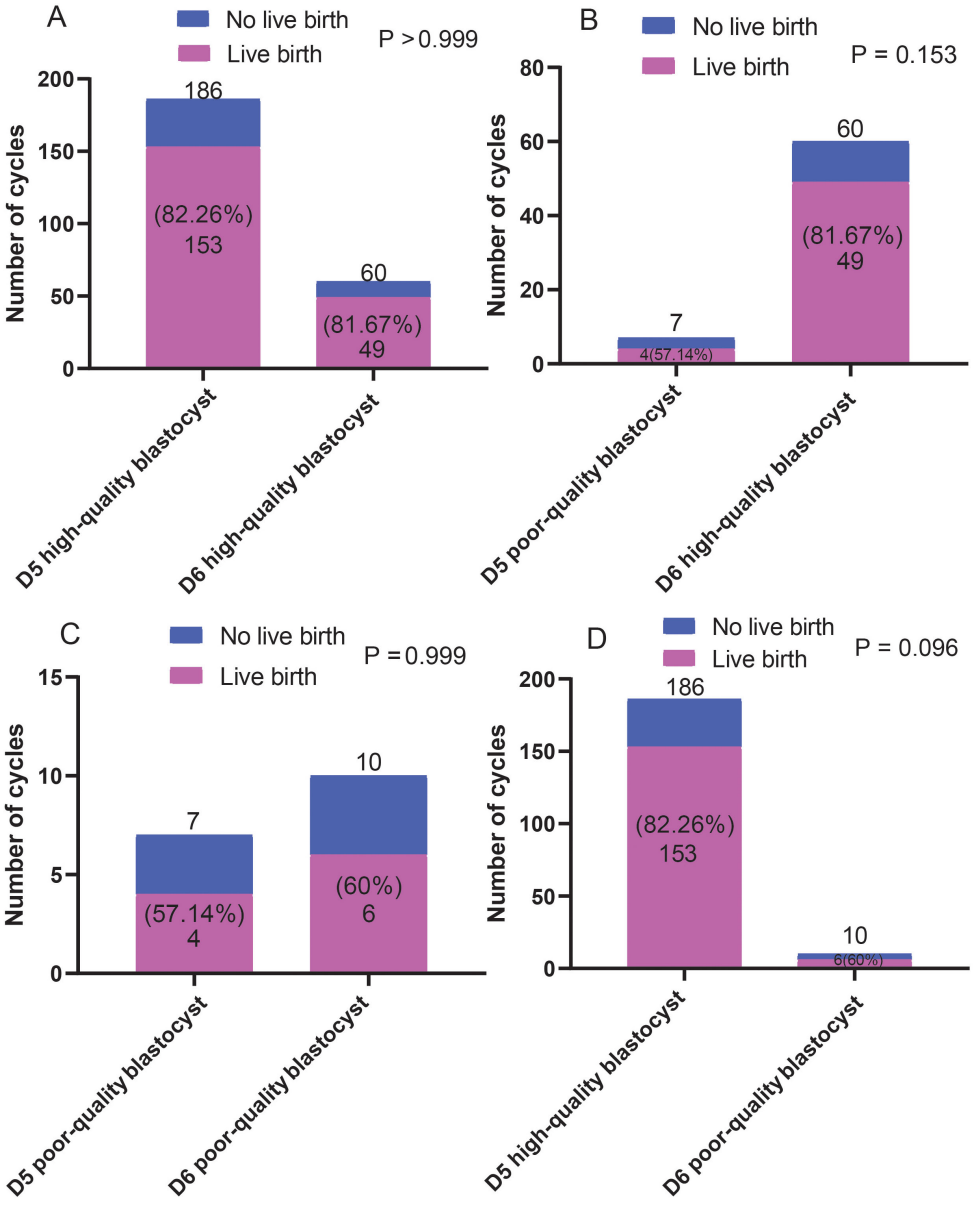
Comparison of the live birth ratios **(A-D)** between patients who received blastocyst transfer of different quality on D5 and D6. Fisher’s exact test was used for statistical analysis.

The effects of blastocysts of D5 and D6 on pregnancy outcomes were compared. We found no differences in pregnancy rates between the D5- and D6-scored A/B blastocyst groups. Similarly, no differences in pregnancy rates were observed between patients who received D5/D6-scored C blastocysts. However, D5-scored A/B blastocyst transfer resulted in a significantly higher pregnancy rate than D6-scored C blastocyst transfer did (**Figure 4**). However, as shown in **Figure 5**, regardless of whether the biopsy date was D5 or D6, no differences in live birth rates were observed between A/B-scored blastocysts (high-quality blastocysts) and C-scored blastocysts (low-quality blastocysts).

## Discussion

Improving clinical pregnancy rates has long been the primary focus of reproductive research. The physical conditions of women and the quality of the embryo are essential factors for successful embryo implantation, and our findings from 428 PGT cycles showed that patient age, BMI, and endometrial thickness are important factors that affect clinical outcomes.

Modern assisted reproductive medicine has progressively implemented an elective single-embryo transfer strategy worldwide to reduce complications related to multiple pregnancies. The main challenge of this strategy is selecting a single embryo with the highest potential for success. Morphological grading criteria are still the most widely used method of embryo selection, but pregnancy outcomes are not satisfactory, and this is a limited predictor of implantation success (14, 17–19). The PGT strategy has proven to be an effective selection tool for increasing the rate of implantation and reducing the rate of pregnancy loss (20). However, research on methods to further improve the success rate of PGT remains relatively limited. The developmental days and quality of embryos are critical in determining whether frozen-thawed blastocyst transfer can lead to clinical pregnancy (21). Therefore, D5 and D6 blastocyst biopsies may have different effects on embryo implantation and clinical outcomes (22). Our study showed that the clinical pregnancy rate of the D5 blastocyst group was significantly higher than that of the D6 blastocyst group over 428 PGT cycles (*P*=0.004), which is also consistent with the findings of previous studies. Kovalevsky et al. showed that the clinical pregnancy rate of D5 blastocysts was significantly higher than that of D6 blastocysts (23). Yu et al. (24) reported similar findings, showing that D5 blastocysts exhibited enhanced transfer success rates and lower miscarriage rates than D6 blastocysts. However, our study found no difference in live birth rates between D5 and D6 blastocysts, and no difference in clinical pregnancy rates and live birth rates between D5-scored A/B blastocysts and D6-scored A/B blastocysts was observed, differing from the findings of other studies.

In our study, both D5 and D6 blastocysts were biopsied only when they had fully expanded to stage 4, with a relatively consistent number and integrity of cells obtained. Nevertheless, Yu et al. (24) reported that D5 blastocysts yielded a lower cell number and integrity than D6 blastocysts. Therefore, this may explain why we observed no difference in live birth rates between the D5 and D6 blastocyst groups. Notably, although the degrees of blastocyst expansion were similar, the clinical pregnancy rate of D5 blastocysts was significantly higher than that of D6 blastocysts.

In this study, we compared the effects of different quality D5 and D6 blastocysts on pregnancy and live birth rates. D5-scored A/B blastocysts resulted in a higher pregnancy rate than that of D6-scored A/B blastocysts; however, live birth rates did not differ significantly between D5-scored A/B blastocysts and D6-scored A/B blastocysts. Similarly, no significant differences were observed in the pregnancy and live birth rates between D5-scored C blastocysts and D6-scored C blastocysts. However, the pregnancy rates of D5-scored A/B blastocysts were significantly higher than those of D6-scored C blastocysts. These findings suggest that differences in embryo quality due to different blastocyst grades on the blastocyst biopsy date D5 or D6 only affect the pregnancy success rate.

This study has several limitations. First, this was a single-center retrospective study. Second, we could not exclude the possibility of residual confounding, and the sample size for this study was relatively small, especially the number of embryos that scored C on D5 and D6. We will increase the sample size in future research to overcome this limitation.

## Conclusion

In this study, we developed a prediction model and identified the factors affecting pregnancy outcomes. To achieve better clinical outcomes, evaluating embryo selection strategies based on morphology and developmental days is crucial, with PGT playing a significant role. Additionally, patients should adjust their BMI, endometrial receptivity, and endometrial thickness and pattern. This study shows that D5-biopsied blastocysts result in higher pregnancy rates than those of D6-biopsied blastocysts. Although D5-scored A/B blastocysts resulted in higher pregnancy rates than those of D6-scored C blastocysts, no significant difference was observed in live birth rates; therefore, considering blastocyst grading and blastocyst biopsy dates is necessary to achieve optimal clinical outcomes in patients undergoing PGT.

## Data Availability

The original contributions presented in the study are included in the article/**Supplementary Material**. Further inquiries can be directed to the corresponding author.
