# Supplementary material for: Impact of blastocyst grading and blastocyst biopsy dates on the clinical outcomes of patients undergoing preimplantation genetic testing
Source: Front Endocrinol (Lausanne). 2024 Sep 20;15:1427922. doi: 10.3389/fendo.2024.1427922 (PMC11449714; doi:10.3389/fendo.2024.1427922)
Supplement: Supplementary file 1 [file Table1.doc]

Supplementary table:The number of couples in each category of the three indications of PGT

|  | Category | Number |
| --- | --- | --- |
| PGT-A  (241) | AMA | 93 |
|  | RIF | 21 |
|  | RPL | 127 |
| PGT-SR  (173) | Translocation | 121 |
|  | Inversion | 50 |
|  | 47,XXX | 2 |
| PGT-M  (14) | Hemophilia | 3 |
|  | Ichthyosis vulgaris | 1 |
|  | Duchenne muscular dystrophy | 2 |
|  | Carrier of the deafness-associated GJB2 gene | 5 |
|  | Congenital muscular dystrophy | 1 |
|  | Spinal muscular atrophy | 2 |

PGT-A：Preimplantation Genetic Testing for Aneuploidies

PGT-SR：Preimplantation Genetic Testing for Structural Rearrangements

PGT-M：Preimplantation Genetic Testing for Monogenic/Single Gene Disorders

AMA: advanced maternal age , defined as ≥37 years

RIF: repeated implantation failure,usually failure after three or more transfers of high quality embryos

RPL: history of recurrent pregnancy loss
